# Levels of Tuberculosis Treatment Adherence among Sputum Smear Positive Pulmonary Tuberculosis Patients Attending Care at Zomba Central Hospital, Southern Malawi, 2007–2008

**DOI:** 10.1371/journal.pone.0063050

**Published:** 2013-05-28

**Authors:** Tobias Chirwa, Peter Nyasulu, Esnat Chirwa, Akeem Ketlogetswe, George Bello, Isiah Dambe, Dennis Ndalama, Martias Joshua

**Affiliations:** 1 Division of Epidemiology and Biostatistics, School of Public Health, Faculty of Health Sciences, University of the Witwatersrand (Wits), Johannesburg, South Africa; 2 Mathematical Sciences Department, Chancellor College, University of Malawi, Zomba, Malawi; 3 Ministry of Health, National TB Control Programme, Lilongwe, Malawi; 4 Research for Equity and Community Health (REACH) Trust, Lilongwe, Malawi; 5 Ministry of Health, Zomba Central Hospital, Zomba, Malawi; Institut de Pharmacologie et de Biologie Structurale, France

## Abstract

**Background:**

Despite great efforts to control Tuberculosis (TB), progress is compromised by low adherence to medication, leading to prolonged duration of infectiousness and continued transmission. Investigating low adherence is of high importance from TB programmatic perspective. Though data on actual days of missed treatment exist, the effect of such on TB cure rates has not been investigated.

**Methods:**

TB operational research data were extracted for smear-positive pulmonary TB patients registered at Zomba Central hospital, Malawi from January 2007 to December 2008.

**Results:**

Of the 524 patients, 302 (57.6%) were males and 340 (64.9%) fully adhered to treatment. Excluding 5 individuals with missing data on cure, four hundred and eighty-one (92.7%) were cured of TB, and of these 162 (33.7%) missed at least one day of treatment. Respectively, 49/64 (76.6%) and 71/76 (93.4%) of those who missed treatment in the intensive and continuation phases were cured of TB (p = 0.005). The adjusted logistic regression analysis showed that those who missed 15–29 days of treatment (OR = 0.04, 95% CI: 0.01, 0.14) were less likely to be cured of TB compared with those who fully adhered.

**Conclusion:**

Treatment non-adherence was high and was observed even within the first 2 months of treatment. Thus, even at an earlier critical stage of treatment, simple algorithms need to be developed to identify and monitor patients at higher risk of non-adherence. Efforts on treatment compliance counselling should focus on enhanced counselling to improve adherence during the intensive treatment phase.

## Background

### TB burden

Tuberculosis (TB) causes a heavy burden of morbidity and mortality, especially in developing countries. An estimated 8.8 million new cases of TB were reported globally with 1.1 million and 0.35 million deaths occurring among HIV-negative and HIV-associated individuals [1] respectively. Global TB report for 2009 estimated that 48,144 new cases of TB occurred in Malawi, a country with approximately 14 million people. Sixty-eight percent of these new cases were due to HIV infection. In recent years there has been a rise in treatment success of up to 78%, however, this still falls below the WHO target point of 85% [1], [2], [3].

### TB treatment

Currently in Malawi standard TB treatment regimens are based on a combination of streptomycin, isoniazid, rifampicin, pyrazinamide and ethambutol. Such combination has shown to be effective in most of cases [4], [5]. During the first two weeks of the intensive phase of treatment, newly diagnosed TB patients receive daily dose of TB treatment while hospitalized. For the remaining six weeks of the intensive phase, patients continue taking their medication following the DOTS (Directly Observed Treatment, Short course) option, either while still in hospital if too ill or in their communities. This treatment modality is different for central hospitals where patients take their TB medication on ambulatory basis from the day they register for TB treatment throughout the intensive phase [3], [4], [5]. In the four months continuation phase, patients take TB medication under a preferred DOT option. Patients collect their monthly supply of drugs from health facilities nearest to them within their catchment area.

### HIV co-infection and treatment adherence

The increase in TB in the communities has been attributed to human immunodeficiency virus (HIV) co-infection as low immune function increases the risk of TB acquisition or reactivation of latent TB. Increased TB prevalence can also be attributed to increased contact with untreated family/household contacts of a primary smear positive TB case [6], [7]. Circulating doses of TB bacilli in the community certainly increases if there is a large pool of untreated TB cases or diagnosed cases that are not adherent to TB treatment [8], [9], [10]. Poor adherence to TB treatment impacts negatively on clinical management and control of tuberculosis as effectiveness of TB medications gets compromised, particularly in resource poor settings [11], [12], [13], [14], [15]. Despite WHO’s efforts of enhancing DOTS strategy for TB treatment [3], often times, patients have discontinued the medication before the completion of the treatment duration, creating a suitable environment for TB relapse as well as emergence of multidrug resistance (MDR) to the standard TB medications [15]–[16].

### Extent of treatment non-adherence

Previous studies done in Africa and Asia [14], [16], [17], [18], [19], [20], [21] have shown that since TB treatment is taken over a longer period of time, this long duration of TB medication might be negatively influencing treatment adherence, leading to low cure rates and high TB-associated mortality. A study by Rocha et al [22] showed that treatment non-adherence was significantly associated with unfavourable outcome (death or no TB cure). In Malawi, the TB treatment default rate ranges from 3% to 16% [5], [23] and is on the increase due to concomitant use of antiretroviral treatment among those with TB/HIV co-infection [24], [25], [26]. Thus, apart from investigating the extent of treatment adherence assessing measures to improve treatment adherence is essential.

### Interventions to improve adherence

Studies that have implemented interventions have shown that good treatment adherence can lead to complete cure from TB. A cluster randomized trial in Senegal found that intensive strategy of treatment monitoring and education led to improved adherence to medications and improved outcomes among TB cases [27], even in the presence of HIV infection [28]. Although Malawi has high prevalence of TB/HIV co-infection [29] associated with poor treatment outcomes, studies on adherence showed improved TB cure rates [23], [30]. However such studies only focused on the impact of adherence during the intensive phase of TB treatment.

### Definition for treatment adherence

Most studies investigating treatment adherence so far were based on the WHO case definition of treatment default. The WHO defines a TB treatment defaulter as a patient who interrupted treatment for two consecutive months or more [31]. However, this definition does not take into account the actual levels of treatment adherence of such patients and considers treatment adherence as a binary variable (i.e. whether someone misses treatment or not) without necessarily considering the actual days of missed TB medications. Although WHO training modules [32] have clear guidelines on how to address treatment interruptions of less than two months and how to compensate for missed doses, this is rarely implemented. This approach was probably a way of simplifying treatment adherence monitoring. Despite the fact that data on missed dosages are available from hospital records, such information has not been fully utilized to investigate the impact of non-adherence to TB medication on cure.

This study has documented, using available operational data, the actual days of missed treatment during the intensive as well as continuation phase of TB treatment. The main objective of the study was to estimate cure rates. In addition, we also investigated whether there is an association between varying levels of adherence and TB cure rates among smear positive pulmonary TB patients after treatment.

## Methodology

### Study design and setting

This was a descriptive study of patients aged 18 years and above admitted with a diagnosis of smear-positive pulmonary TB at Zomba Central Hospital in south-eastern region of Malawi. The study utilized routine operational TB data.

### Sample Size

This was a secondary data analysis of operational research data and the study utilised all relevant records for 524 smear-positive pulmonary TB patients registered for TB treatment between January 2007 and December 2008. Sample size was obtained using Epi Info version 3.5.1. Considering that we want to estimate cure rates, a minimum of 310 patients was required, assuming a true cure rate of 73% and we wanted an estimate to within 5% precision with 95% confidence. The cure rate was obtained from 2003 national TB quarterly reports from Hospitals which included Zomba Central Hospital where this study was done. We therefore conclude that the data extracted from relevant records was thus sufficient to estimate cure rates and explore associations with levels of adherence.

### Data Collection

Operational data for TB patients were extracted from the patient’s medical records by trained research assistants under the supervision of the District TB Officer. The sources of operational data included patients’ treatment cards, TB registers, ART registers and TB drug balance recording books. Data extracted included age and sex; sputum smear microscopic results, treatment course (phase of TB treatment), treatment cure at the end of the treatment period (or outcome), HIV status, and antiretroviral (ART) medication history. Relevant data were extracted onto a data collection sheet. Treatment monitoring and adherence data were accessed from treatment cards whose continuation phase information on number of days a patient missed treatment was sourced from patients themselves. Treatment non-adherence was defined as the actual number of days a patient missed TB treatment over the prescribed treatment period. Non-adherence was recorded on a monthly basis from daily record sheets of treatment uptake. Sputum examination was done at treatment initiation and thereafter monthly for the 6 months treatment duration. TB cure was defined as sputum smear negativity at the end of treatment regimen. Although the definition used for this study is slightly varied from the WHO definition which requires an additional sputum smear negative in another occasion during the course of treatment, the cure numbers are not very different as we observed high consecutive sputum smear negativity from two months onwards.

### Data management and analysis

Since data were extracted from patients’ records and recorded on the study forms, data were doubly entered in EPI INFO (Version 3.5.1 CDC, Atlanta, USA) cleaned and analysed using Stata version 10 (StataCorp Limited, College Station, Texas, USA). For purposes of analysis, treatment non-adherence was further categorized into 15 days interval (0, 1–14 days; 15–29 days; and ≥ 30 days).

Frequency tabulations were produced for demographic characteristics (age and gender); number of actual days treatment was taken, HIV status and treatment outcomes based on sputum smear results. To investigate whether significant associations exist between demographic, clinical factors and treatment outcome (TB cure), a Chi-Square test was used. Factors whose p-values showed significant association with the outcome at a conservative 20% significance level were included in the multiple logistic regression models using a stepwise approach based on standard use [33]. The regression analysis was used to determine factors associated with treatment outcome.

### Ethical Approval

The study was approved by the Human Research Ethics Committee of the University of the Witwatersrand (ethical clearance number M090939) and Malawi Health Sciences Research Committees (approval number 684).

Consent to use medical records of patients attending treatment at the Zomba Central Hospital, TB clinic was obtained from the Hospital Administration who has the jurisdiction to grant or deny access to use of patient operational records for research as we had done. The records are in form of hard copy TB cards and folders that stores all other patient details. Data was extracted from the hard copy records and entered and analysed anonymously. This is one of the conditions set by the national ethics board as and when the medical records are used as such no patient consent is required. The letter from the Hospital Administration granting permission to use the data was submitted as supporting documents. These institutional ethics committees therefore waived the need for written informed consent from the participants based on the permission letter from the Hospital Administrator.

## Results

### Demographic and clinical characteristics of patients

Table 1 shows demographic and clinical characteristics of the patients. The study included 524 TB patients of whom 302 (57.6%) were males. The mean age of the patients was 36.0 (standard deviation (SD): 12.4) years, with 347 (68.3%) being young adults aged less than 40 years. Four hundred and sixty (87.8%) were new cases of TB, 60 (11.4%) relapse cases and 4 (0.8%) were treatment failures. Two hundred and seventy four (65%) of the patients were HIV positive, of these only 78 (29%) were on ART.

**Table 1:** Characteristics of 524 smear-positive pulmonary TB patients registered for TB treatment from January 2007 to December 2008 at Zomba Central Hospital, Malawi.

Factor	Category	Frequency	
		n	%
**Gender**	**Male**	302	57.6
	**Female**	222	42.4
**Age Group (in years)**	**<30**	181	35.6
	**30–39**	166	32.7
	**40–49**	79	15.6
	**≥50**	82	16.1
**DOTs option**	**Guardian**	397	76.5
	**Hospital**	91	17.5
	**Health centre**	31	6.0
**Patient category**	**New case**	460	87.8
	**Relapse**	60	11.4
	**Failure**	4	0.8
**HIV Status**	**Negative**	147	34.9
	**Positive**	274	65.1
**Actual days treatment missed**	**None**	340	64.9
	**< 15**	159	30.3
	**15–29**	20	3.8
	**≥ 30**	5	1.0
**Missed treatment in phase**	**None**	340	64.9
	**Continuation**	77	14.7
	**Intensive**	64	12.2
	**Both phases**	43	8.2
**Treatment outcome**	**Cured**	481	92.7
	**Not cured**	38	7.3

Overall, 184 (35.1 %) patients did not fully adhere to TB treatment. Of these, 159/184 (86.4%) missed less than 15 days of treatment and 43 (23.4%) patients missed at least one day of treatment in both the intensive and continuation treatment phases.

Five of the 524 individuals had missing data on cure from TB. Overall, 481 (92.7%) patients were cured from TB and 162 (33.7%) of these missed at least one day of treatment. The majority of the patients on treatment were monitored by their guardians, 397 (76.5%), few patients, 91 (17.5%), were hospitalised patients and only 31 (6.0%) were monitored at a health centre facility.

### Levels of treatment non-adherence

Table 2 shows levels of treatment non-adherence within intensive and continuation treatment phase. Within the intensive phase, the proportion of patients who had missed at least one day of treatment was 107/524 (20.4%), with 93 (17.8%) missing between 1–14 days and 14 (2.7%) patients missing 15–29 days of treatment.

**Table 2:** Frequency distribution of levels of non-adherence to treatment (in days) within intensive and continuation treatment phase (n = 524).

Factor		Treatment Phase	
		Intensive	Continuation
**Mean number (SD) of days of missed treatment on a monthly basis**	**1^st^ month**	4.72 (7.5)	-
	**2^nd^ month**	4.12 (5.7)	-
	**3^rd^ month**		5.47 (10.2)
	**4^th^ month**		3.90 (6.3)
	**5^th^ month**		4.22 (5.2)
	**6^th^ month**		3.26 (4.5)
**Default Status – WHO definition**	**Yes**	-	1 (0.2%)
	**No**	524 (100%)	523 (99.8%)
**Non-adherence levels**	**0 days**	417 (79.6%)	404 (77.1%)
	**<15 days**	93 (17.8%)	108 (20.6%)
	**15–29 days**	14 (2.7%)	7 (1.3%)
	**30–44 days**	-	4 (0.8%)
	**≥45 days**	-	1 (0.2%)

During the continuation phase, 120 (22.9%) patients missed treatment of which 108 (20.6%) missed less than 15 days; 7 (1.3%) missed 15–29 days and 5 missed more than 30 days of treatment.

There were 52% (56/107) and 63% (75/120) male patients who missed treatment in the intensive phase and continuation phases respectively. Among those who missed treatment in the intensive phase, 91 (85.1%) were cured compared to 113 (95.0%) in the continuation phase. To avoid double counting, a repeat analysis showed that among those who only missed the intensive phase, 49/64 (76.6%) were cured compared to 71/76 (93.4%) in the continuation phase (p = 0.005). For those who missed at least one day of treatment in both phases, 42/43 (97.7%) were cured of TB.

### Factors associated with cure from TB

Table 3 presents results of unadjusted and adjusted logistic regression models investigating the association between factors and cure from TB.

**Table 3:** Univariate and multiple logistic regression analysis of factors associated with cure from TB (n = 519).

Factor	Level	Proportion cured	Univariate Regression		Multiple Regression	
		Freq/n (%)	OR	95% CI	OR	95% CI
**Gender**	**Male**	277/299 (92.6)	1		1	
	**Female**	204/220 (92.7)	1.01	0.52–1.98	1.32	0.56–3.11
**Age Group (in years)**	**<30**	169/180 (93.9)	1		1	
	**30–39**	146/164 (89.0)	0.53	0.24–1.15	0.72	0.25–2.06
	**40–49**	72/79 (91.1)	0.67	0.25–1.80	0.59	0.18–1.92
	**≥50**	78/80 (97.5)	2.54	0.55–11.73	1.09	0.21–5.66
	**Missing**	16/16 (100)				
**Actual days treatment missed**	**None**	319/336 (94.9)	1			
	**< 15**	149/159 (93.7)	0.79	0.36–1.78	0.84	0.34–2.08
	**15–29**	8/19 (42.1)	0.04	0.01–0.11	0.04	0.01–0.14
	**≥ 30**	5/5 (100.0)	-	-	-	-
**^+^Missed Treatment in Intensive Phase**	**No**	390/412 (94.7)	1			
	**Yes**	91/107 (85.1)	0.32	0.16–0.64	-	-
**Missed Treatment in Continuation Phase**	**No**	368/400 (92.0)	1			
	**Yes**	113/119 (95.0)	1.64	0.67–4.02	-	-
**DOTS Option**	**Guardian**	362/393 (92.1)	1			
	**Hospital**	85/91 (93.4)	1.21	0.49–3.00	-	-
	**Health Centre**	29/30 (96.7)	2.48	0.33–18.85	-	-
	**Missing**	5/5 (100)				
**Patient Category**	**New Case**	425/455 (93.4)	1		1	
	**Relapse**	54/60 (90.0)	0.64	0.25–1.60	0.60	0.20–1.84
	**Failure**	2/4 (50.0)	0.07	0.01–0.52	0.09	0.01–1.18
**Whether the patient was on ART**	**Yes**	70/78 (89.7)	1			
	**No**	173/190 (91.1)	1.16	0.48–2.82	-	-
	**Missing**	238/251 (94.8)				
**HIV status**	**Negative**	140/144 (97.2)	1		1	
	**Positive**	247/273 (90.5)	0.27	0.09–0.79	0.35	0.11–1.10
	**Unknown**	94/102 (92.2)				
**Initial smear positivity**	**1+**	127/139 (91.4)	1			
	**2+**	113/124 (91.1)	0.97	0.41–2.29	-	-
	**3+**	193/205 (94.2)	1.52	0.66–3.49	-	-
	**Scanty**	44/46 (95.7)	2.08	0.45–9.65	-	-
	**Missing**	4/5 (80.0)				

**Note:** **^+^** - was not included in the adjusted model as it is highly correlated with actual number of days missed treatment.

In the univariate analysis, factors that were significantly associated with cure from TB included missing treatment in the intensive phase, whether a patient was a previously treatment failure case, HIV status and actual number of days when treatment was not taken. These were considered further in the adjusted analysis. In the adjusted analysis, patients who missed less than 15 days and 15–29 days of treatment were less likely to be cured (OR = 0.84, 95% CI: 0.34–2.08 and OR = 0.04, 95% CI: 0.01, 0.14 respectively) compared with those who fully adhered. Although not significant, the odds ratio for patients who missed treatment in the intensive phase was 0.58 (95% CI: 0.16–2.02). Previously failed treatment cases (OR = 0.07, 95% CI: 0.01–0.52) and HIV positive cases (OR = 0.27, 95% CI: 0.09, 0.79) were also less likely to be cured compared to HIV negative TB cases.

## Discussion

Even though it is biologically plausible that good adherence to treatment leads to improved cure rates and minimizes development of drug resistance, there has been limited work investigating the actual levels of treatment adherence as we have done. Such adherence patterns and how they arise are described elsewhere [34] in form of missing data patterns. This paper has documented the treatment adherence levels in the intensive and continuation phases focusing on their effects on cure from TB at the end of the treatment period.

The study has shown that, in general, the chances of cure are higher among new TB cases compared with relapse or treatment failed case and that TB cure rate decreases with an increase in number of days of treatment missed i.e. from 95% in those who fully adhered to 42% among those who missed 15–29 days of treatment. The 100% cure rate in those who missed treatment for more than 30 days might partly be attributed to small numbers. Ideally, we require a 100% level of treatment adherence to achieve optimum cure, however, this is often not achieved in current clinical practices. Thus, to ensure adequate cure rate from TB, we need to find ways of advocating for complete adherence to TB treatment in Malawi. As with antiretroviral medications for HIV/AIDS, TB therapy requires high compliance to medication to facilitate cure [31].

The effect of adherence on cure from TB may also depend on the treatment phase where such non-adherence occurred. The study has shown that although the differences were not significant in the adjusted analysis and could be due to chance, cure rate was lower among those who missed treatment in the intensive phase (77%) compared to the continuation phase (93%). Thus, the estimates point to the fact that adherence to treatment in the intensive phase is critical for cure from TB. This confirms findings from another study [21] that looked only at the intensive phase. Considering that the whole mark of TB control rests in sterilising patients within the first two months [3], [5], this study highlights the importance of treatment adherence in the intensive phase.

It is interesting to note that should our study have used the WHO case definition for default status [1], [3], [5], we would only have identified one case with poor adherence to treatment. This therefore throws some light that most investigators might likely miss such an important factor that has an impact on cure among TB patients. This study has shown that even if a TB patient was to miss treatment for a few days particularly during the intensive phase, such a situation would compromise TB cure among those on TB medication. Missing treatment during the intensive phase is rather a misnomer as the common expectation is that during this intensive phase, patients on TB treatment are closely monitored [3], [4]. Previous reports have shown that some patients are allowed to take medication from home during the intensive phase, especially in urban settings. This might influence poor adherence and lead to failed treatment and continued infectiousness [35].

Considering that this is based on operational data, it is not without its limitations. Information on drug resistance was not available which could explain the low cure rates seen among relapse and failure cases. Further, the treatment adherence levels were based on reported information from patients, especially in the continuation phase where the guardian was responsible to ensure that TB drugs were taken. In such cases, a prospective study with patients strictly monitored would be more appropriate.

## Conclusion

We found high numbers of individuals who did not adhere to treatment. Simple algorithms utilizing patient characteristics can be developed for use in TB clinics in order to identify and actively monitor patients who are more likely not to adhere to TB treatment.

The critical period for cure from TB is the intensive phase and efforts to advocate full adherence need to be emphasised in Malawi if we are to control tuberculosis. Novel ideas such as use of latest IT mobile technology can be explored as a way of reinforcing treatment adherence particularly during the intensive treatment phase of TB treatment.
